# Initiation of the First Preventive Health and Screening Outpatient Department in a Tertiary Teaching Hospital in India

**DOI:** 10.7759/cureus.38115

**Published:** 2023-04-25

**Authors:** Jugal Kishore, Sunanda Gupta, Pratima Gedam

**Affiliations:** 1 Department of Community Medicine, Vardhman Mahavir Medical College and Safdarjung Hospital, New Delhi, IND

**Keywords:** outpatient department (opd), screening, lifestyle modifications, primary prevention, health promotion, salutogenesis, lifestyle changes, non-communicable diseases, preventive medicine

## Abstract

Background

The Preventive Health and Screening Outpatient Department (OPD) was started in Vardhman Mahavir Medical College and Safdarjung Hospital, Delhi, India with the vision of promoting health (primordial and primary prevention), counseling, screening, early diagnosis, and treatment and referral services (secondary prevention). The objective of the study is to describe the process of establishing the Preventive Health and Screening OPD in a tertiary hospital in Delhi and illustrate the functioning of the newly established OPD.

Methodology

This study is based on observation of the day-to-day functioning of the OPD, record checking of registers, and reviewing the records of the hospital registration system. Here, we describe the functioning of the OPD from its initiation in October 2021 until December 2022.

Results

The routine services provided at the OPD include health promotion and education, especially focusing on non-communicable diseases, screening, diagnosis, treatment, lifestyle counseling; general OPD services; growth monitoring and counseling; group discussion for harms of tobacco usage; counseling for tobacco cessation, hepatitis B, and dT vaccination; group counseling for antenatal women; and screening for breast cancer. A few events such as the breast cancer screening camp and the non-communicable disease screening camp were also conducted under the purview of the new OPD. Such OPDs are the need of the hour for the provision of comprehensive healthcare, including promotive and preventive healthcare, along with curative health services, at tertiary healthcare levels.

Conclusions

Healthcare services are incomplete without the preventive, promotive, and screening components of healthcare. For mainstreaming health promotion and preventive healthcare, Preventive Health and Screening OPDs are essential at hospitals. The benefits of prevention extend beyond managing chronic diseases and longer lives.

## Introduction

Three levels of prevention and five levels of preventive interventions, as promoted by Leavell and Clark, are taught in all healthcare institutions under the concept of health and disease [1]. “Prevention is better than cure” is the most common saying in all cultures. However, the focus of healthcare institutions is often ignoring primordial and primary prevention. Why should we wait till it is too late to regain well-being? We should actively strive to gain health. It is better explained by a medical sociology professor, Aaron Antonovsky, in his 1979 book Health, Stress and Coping, who coined the term Salutogenesis [2]. It is the study of human health and well-being, rather than a focus on factors that cause disease (pathogenesis). Preventive care “has the aim of preventing disease or its consequences. It includes health care programs aimed at warding off illnesses, early detection of disease, and inhibiting further deterioration of the body” [3]. On the other hand, preventive medicine is “the branch of medicine dealing with the prevention of disease and the maintenance of good health practices” through primordial prevention, health promotion, and specific protection [3]. The World Health Organization (WHO) defines prevention as “approaches and activities aimed at reducing the likelihood that a disease or disorder will affect an individual, interrupting or slowing the progress of the disorder or reducing disability” [4]. The key role of a community medicine specialist has been recognized as the identification of determinants (direct and indirect), influencing health and diseases, and the organization of healthcare services to attain the optimal quality of health [5]. The functions of a physician trained in community medicine expand beyond the five levels of prevention, viz., health promotion, specific protection, early diagnosis and prompt treatment, disability limitation, and rehabilitation [6].

Evidence has proved that investment in public health is more efficient and effective than an emphasis on curative and rehabilitative services [7-9]. The health policy of India aims at achieving improvement of health status through concerted policy action in all sectors and expanding preventive, promotive, curative, palliative, and rehabilitative services provided through the public health sector with a focus on quality [10]. Despite the emphasis, preventive and promotive healthcare in India are the most neglected areas among health services [11]. Currently, the health system in India is largely focused on the treatment of illness and disease.

The objective of the study is to describe the process of establishing a unique outpatient department (OPD), the Preventive Health, and Screening Outpatient Department, in a tertiary hospital in Delhi, and illustrate the functioning of the newly established OPD. The Preventive Health and Screening OPD was started in Vardhman Mahavir Medical College and Safdarjung Hospital by the Department for Community Medicine for promoting health and preventive measures at the tertiary healthcare level.

## Materials and methods

The present study is a descriptive account involving meticulous observation and review of hospital records. The initiation process of the OPD has been delineated through a comprehensive analysis of the proposal (for the establishment of the OPD) and minutes of the meeting register. Furthermore, data collection was conducted through daily observations of the OPD’s operational functioning. In addition, hospital registration reports spanning a period of 15 months, from October 2021 to December 2022, were thoroughly reviewed for data analysis and reporting purposes.

## Results

Establishment of the Preventive Health and Screening Outpatient Department

The inception of the Preventive Health and Screening OPD at Vardhman Mahavir Medical College and Safdarjung Hospital, Delhi, was formulated with the vision of promoting health (primordial and primary prevention), screening, early diagnosis and treatment (secondary prevention), counseling, and referral services. The intention of establishing such an OPD was set in motion by the Department of Community Medicine. The proposal was discussed among the faculty of the department. Debates and discussions regarding the selection of services, functioning of the OPD, the utility of such an OPD, the demand of the OPD, and logistics requirements followed. The priority areas were marked, and the services list was finalized for the OPD. After repeated persuasion, with the support of the Medical Superintendent and the Ministry of Health and Family Welfare, Government of India, the OPD was started in the new OPD building of Vardhman Mahavir Medical College and Safdarjung Hospital, Delhi, in September 2021. The staff posted in the OPD is listed in Table 1.

**Table 1:** Staff posted at the Preventive Health and Screening Outpatient Department.

Staff	Number
Faculty consultants	7
Senior residents (rotational basis)	1–2
Postgraduate trainees (on a rotational basis)	2–4
Junior residents	1–2
Interns (on a rational basis)	2–3
Public health nurse	1
Health educator	1
Lab technician	1
Medical social worker	1
Sanitation attendant/Nursing attendant	1

**Figure 1:**
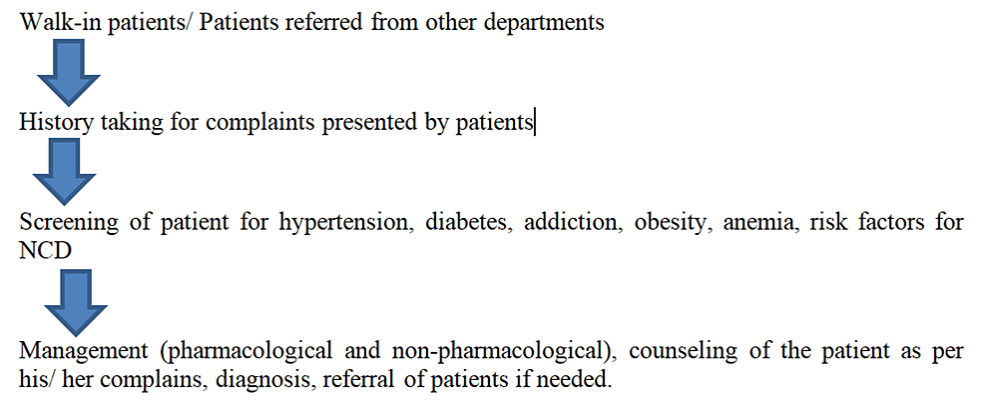
Flow of outpatient department services. NCD: non-communicable disease

The OPD has delivered health care to its beneficiaries for more than 15 months until December 2022. This has been mediated by conducting health education sessions, group and individual counseling of beneficiaries, screening of high-risk groups, and, per demand, diagnosis and treatment of common illnesses, non-pharmacological interventions, lifestyle counseling, adult vaccination, and referral to other departments. Citizen charters were put up at different locations in the hospital to inform about the new OPD and its services. Beneficiaries in the OPD were either walk-in patients, referred from other departments or from the field practice areas, and referred cases from peripheral clinics of the Department of Community Medicine. The flow of OPD services is illustrated in Figure 1.

Non-communicable disease screening, diagnosis, and treatment and lifestyle counseling

Opportunistic screening for diabetes, hypertension, oral cancer, and breast cancer was conducted for all walk-in patients aged 30 and above. Under the purview of occupational health checkups, all security guards employed in the hospital were screened for diabetes, hypertension, and oral cancer. During the screening, a history of diet, medication, and addiction was also obtained. The guidelines followed for the screening and management of the beneficiaries were in accordance with the guidelines issued under the National Programme for Prevention and Control of Cancer, Diabetes, Cardiovascular Diseases and Stroke, Ministry of Health and Family Welfare, India. All beneficiaries with elevated blood pressure and blood glucose level were followed up and provided treatment and counseling for lifestyle modification. All patients were explained about diet, exercise, frequency of follow-up visits, the importance of treatment adherence, blood investigations, possible disease complications, and ways to prevent the complications. If required, patients were referred to other specialized departments.

General outpatient services (management and referral)

Patients with common illnesses were treated according to standard treatment protocols. For chronic non-communicable diseases such as diabetes, hypertension, and osteoarthritis, lifestyle modification was emphasized, along with pharmacological treatment. Patients were also explained the importance of treatment compliance and regular follow-up visits. Opportunistically, patients were educated about the dangers of antibiotic abuse and were discouraged from the use of over-the-counter antibiotics. For all patients, non-pharmacological interventions were equally highlighted as the pharmacological part of the treatment during the counseling.

Group discussion for harms of tobacco usage and counseling for tobacco cessation

All security guards, sanitation workers, and nursing attendants in the hospital were provided health education on the harms of tobacco usage in group counseling sessions. All tobacco users, identified during non-communicable disease screening, were registered for tobacco cessation. All counseling sessions were conducted with the help of a validated flipchart. Patients were referred to the Department of Psychiatry if indicated.

Adult vaccination center

A center was established for adult vaccination where hepatitis B and dT vaccinations were regularly provided at the OPD. First-degree relatives of hepatitis B patients were counseled about the ways of transmission and prevention of hepatitis B. All hospital staff was encouraged to get regular doses of hepatitis B and flu vaccines according to schedule.

Health promotion and health education

Resident doctors and interns posted at the OPD conducted regular health education sessions about relevant topics using flipcharts at the OPD premises and the waiting areas of the hospital.

Group counseling for antenatal women

Group counseling sessions for antenatal women were conducted in the OPD, with the help of a PowerPoint presentation. The beneficiaries were explained about medication intake, diet, rest, contraindications during pregnancy, birth preparedness, breastfeeding, postnatal care, and family planning. At the end of the counseling, the queries of the beneficiaries were addressed. All antenatal women were screened for any anomaly of the nipple which might cause difficulty in breastfeeding.

The well women clinic

All women above 30 years of age were screened for breast and cervical cancer. This service was conducted in coordination with the Department of Obstetrics and Gynaecology. The beneficiaries were also taught about self-breast examination. Beneficiaries who screened positive for breast lumps were referred to the Department of Surgery.

Growth monitoring and counseling

A total of 33 children were found to be malnourished after screening in 15 months. They were screened for anemia and other common illnesses. Along with treatment, their attendants were counseled on the diet and care of the children.

After 15 months of functioning of the OPD, the total number of beneficiaries of different services is presented in Table 2.

**Table 2:** Total number of beneficiaries at the Preventive Health and Screening Outpatient Department.

Services	Number of beneficiaries
Number of beneficiaries screened for non-communicable diseases	939
Number for beneficiaries counseled for antenatal, postpartum, and newborn care	39
Number of beneficiaries in the group discussion for harmful effects of tobacco	245
Number of under-five children screened positive for malnutrition	33
Number of doses injected for hepatitis B vaccination	1,351
Number of beneficiaries for general outpatient department services	1,024

The Preventive Health and Screening OPD aimed at achieving health in a holistic way. Hence, apart from the above-mentioned services, a few special events were conducted by the Department of Community Medicine, at the OPD premises.

In November 2021, a blood sugar screening camp was conducted on the occasion of World Diabetes Day. A total of 214 people were tested. Among them, 32 individuals were found to be diabetic who were unaware of their status. In total, 29 people were found to have uncontrolled blood glucose levels. All diabetic patients were treated and informed about the dietary and lifestyle changes at the Preventive Health and Screening OPD.

On the occasion of International Women’s Day, the Department of Community Medicine and the Department of Obstetrics and Gynaecology, Vardhman Mahavir Medical College and Safdarjung Hospital, in collaboration with the Association of Obstetricians & Gynaecologists of Delhi conducted Women Cancer Screening Fortnight. A total of 70 women visiting the hospital were screened for cervical (visual inspection under acetic acid examination) and breast cancer (clinical breast examination). Moreover, their knowledge of cervical and breast cancer was assessed. They were educated on the risk factors, symptoms, preventive measures of cervical and breast cancer, and self-breast examination.

On the occasion of International Day of Yoga 2022, the Central Council for Research in Yoga and Naturopathy, Ministry of AYUSH, Government of India along with the Department of Community Medicine, conducted an event with the theme “Yoga for Humanity” at the Preventive Health and Screening OPD. The standard Yoga protocol was demonstrated. The program was attended by around 150 participants comprising MBBS interns, faculty employees, security staff, and nursing staff.

A refresher training session about anti-rabies vaccination was held in the OPD. The participants included nurses, postgraduate trainees, junior residents, and interns. An interactive discussion about the vaccination schedule, indications, and challenges was conducted. Queries of the staff posted at the Rabies Vaccination Clinic were addressed at the end of the session.

## Discussion

Safdarjung Hospital, a 1,531-bedded multi-specialty with an additional 500 emergency beds, is one of the largest tertiary care teaching hospitals in India. It is a Central Government Hospital under the Ministry of Health & Family Welfare, Government of India. The hospital has a monthly OPD attendance of approximately 200,000 which includes patients from all age groups, socioeconomic strata, and disease spectrum from all over India. Patients are mostly accompanied by one or more attendants. This heavy footfall provides an opportunity to cater promotive and preventive care to them within a tertiary care setup. With almost 10,000 patients per working day, there are time constraints faced by treating physicians as well as supporting paramedical staff to provide detailed health education and health promotion advice to patients.

Health education regarding the prevention of diseases, lifestyle modification, and overall health promotion is an important component of disease management and holistic health improvement. Such advice should be given in a detailed manner and should be personalized to the patient’s illness, risk factors, and level of understanding to ensure the proper transfer of information and improve compliance. Such advice assumes greater importance in the context of non-communicable diseases because these diseases are influenced by modifiable lifestyle factors to a great extent. Properly disseminated health information and health promotion advice has the potential to prevent non-communicable diseases, earlier detection, and/or reduce complications of such diseases. In addition, screening for non-communicable diseases is important in the Indian scenario because of the lack of awareness and regular screening. Such screening helps in the earlier detection of non-communicable diseases and ensures better treatment and control while reducing potential complications. Similarly, antenatal care also requires counseling for lifestyle modification apart from medicinal treatment. Growth monitoring of under-five children and advice for defaulters also require individual counseling and guidance.

Preventive care services can promote healthier lifestyles, provide early detection of diseases, and reduce the need for inpatient care services among individuals [12]. A similar finding was reported in another study which reported that investing in preventive care may contribute to reducing subsequent demand for medical care. [13].

The private healthcare sector is presently catering to the increasing demand for preventive healthcare [14-16]. The government healthcare sector in India is yet to respond to this progressive demand for preventive healthcare. The OPDs at the tertiary healthcare level in the public sector are already overburdened with the large patient population [17]. Adding services of preventive healthcare practice and screening (for early detection of disease) and counseling will solve the crisis of the specialist OPDs at tertiary hospitals. The community medicine departments of most teaching hospitals in India engage in primary healthcare services and restrict their activities in community and peripheral healthcare centers. However, there is a strong need for preventive and promotive health and screening OPD at the tertiary healthcare level for comprehensive healthcare provision [18-20]. Studies conducted abroad also support this concept of comprehensive healthcare [21-23]. This is in line with the national drive of Ayushman Bharat which aims to move forward from a selective approach to healthcare to deliver a comprehensive range of services spanning from preventive to promotive healthcare along with curative healthcare services [24]. Hospitals should not only focus on pathogenesis but also start practicing and preaching salutogenesis [25-27]. Healthcare practices embracing all three levels of prevention will pave the way for further quaternary prevention levels beyond the limitations faced today [28].

Our study is not without limitations. Because our study is review and observation-based, it suffers from the inherent problem of incomplete and inconsistent existing data. The study elaborates on a period of the initial 15 months. It is limited by the timeframe of data collection, which may not capture changes or trends that occur outside of the study period.

## Conclusions

Healthcare services are incomplete without the preventive, promotive, and screening components of healthcare. The benefits of prevention extend beyond managing chronic diseases and longer lives. For mainstreaming health promotion and preventive healthcare, Preventive Health and Screening OPDs are essential at hospitals. Tertiary hospitals being the apex centers in our country should attend to their beneficiaries holistically beyond the narrow purview of curative medicinal practice. Research should be encouraged for assessing the feasibility of the implementation of such OPDs, exploring barriers, ways to awareness and demand among the community for the services, and improving the effectiveness of such OPDs.
